# 3D Poly(Lactic Acid) Scaffolds Promote Different Behaviors on Endothelial Progenitors and Adipose-Derived Stromal Cells in Comparison With Standard 2D Cultures

**DOI:** 10.3389/fbioe.2021.700862

**Published:** 2021-09-08

**Authors:** Giuliana Biagini, Alexandra Cristina Senegaglia, Tarciso Pereira, Lucas Freitas Berti, Bruna Hilzendeger Marcon, Marco Augusto Stimamiglio

**Affiliations:** ^1^ Laboratório de Biologia Básica de Células-Tronco, Instituto Carlos Chagas, Fiocruz Paraná, Curitiba, Brazil; ^2^ Núcleo de Tecnologia Celular, Escola de Medicina, Pontifícia Universidade Católica Do Paraná, Curitiba, Brazil; ^3^ Department of Mechanical Engineering, Post Graduate Program in Biomedical Engineering, Universidade Tecnológica Federal do Paraná, Curitiba, Brazil

**Keywords:** mesenchymal stem cells, tissue engineering, 3D scaffold, expanded CD133+ cells, poly(lactic acid)–PLA

## Abstract

Tissue engineering is a branch of regenerative medicine, which comprises the combination of biomaterials, cells and other bioactive molecules to regenerate tissues. Biomaterial scaffolds act as substrate and as physical support for cells and they can also reproduce the extracellular matrix cues. Although tissue engineering applications in cellular therapy tend to focus on the use of specialized cells from particular tissues or stem cells, little attention has been paid to endothelial progenitors, an important cell type in tissue regeneration. We combined 3D printed poly(lactic acid) scaffolds comprising two different pore sizes with human adipose-derived stromal cells (hASCs) and expanded CD133^+^ cells to evaluate how these two cell types respond to the different architectures. hASCs represent an ideal source of cells for tissue engineering applications due to their low immunogenicity, paracrine activity and ability to differentiate. Expanded CD133^+^ cells were isolated from umbilical cord blood and represent a source of endothelial-like cells with angiogenic potential. Fluorescence microscopy and scanning electron microscopy showed that both cell types were able to adhere to the scaffolds and maintain their characteristic morphologies. The porous PLA scaffolds stimulated cell cycle progression of hASCs but led to an arrest in the G1 phase and reduced proliferation of expanded CD133^+^ cells. Also, while hASCs maintained their undifferentiated profile after 7 days of culture on the scaffolds, expanded CD133^+^ cells presented a reduction of the von Willebrand factor (vWF), which affected the cells’ angiogenic potential. We did not observe changes in cell behavior for any of the parameters analyzed between the scaffolds with different pore sizes, but the 3D environment created by the scaffolds had different effects on the cell types tested. Unlike the extensively used mesenchymal stem cell types, the 3D PLA scaffolds led to opposite behaviors of the expanded CD133^+^ cells in terms of cytotoxicity, proliferation and immunophenotype. The results obtained reinforce the importance of studying how different cell types respond to 3D culture systems when considering the scaffold approach for tissue engineering.

## Introduction

As tissue engineering techniques evolve, the need for understanding three-dimensional (3D) microenvironments becomes more pronounced, considering that two-dimensional (2D) cultures are not an ideal model to predict cell behavior in the 3D environment of the biological tissues (Jensen and Teng, 2020). The cues to which cells are exposed in 3D and 2D culture systems are not the same and yield different cell responses (Baker and Chen, 2012; Jensen and Teng, 2020). A 3D system differs from a 2D culture flask mostly due to the fact that the cells can experience a surrounding network in which they are exposed to a gradient of nutrients and to a surface that can present heterogeneous composition and stiffness (Baker and Chen, 2012). The latter aspect is essential because cells respond to matrix stiffness through a mechanism known as mechanotransduction that can dictate cell migration, proliferation and even differentiation (Baker and Chen, 2012; Guimarães et al., 2020).

In the classical approach of tissue engineering, 3D culture is performed on scaffolds made of biomaterials (Gaharwar et al., 2020). In this case, the scaffold acts as a support for cells to proliferate and secrete the extracellular matrix (Abbot and Kaplan, 2016; Gaharwar et al., 2020). To this purpose, the biomaterial must be biocompatible and biodegradable (Abbot and Kaplan, 2016). One biomaterial of great interest for tissue engineering applications is poly(lactic acid) (PLA), mainly due to its excellent biocompatibility and biodegradability (Singhvi et al., 2019). In addition, PLA is a good biomaterial option because it can be produced from renewable sources, it can be easily combined with other biomaterials, and compared to other biomaterials, its processing methods are more straightforward (Casalini et al., 2019).

PLA scaffolds can be produced by different techniques, such as electrospinning (Islami et al., 2018), melt-blown technique (Jenkins et al., 2017; Dzierzkowska et al., 2021) and 3D printing (Almeida et al., 2014; Diomede et al., 2018; Fairag et al., 2019). The processing parameters can be tuned to create several 3D architectures for different applications and induce specific cell responses (Fairag et al., 2019; Dzierzkowska et al., 2021). The most widely used 3D printing technique is fused deposition modeling (FDM), which consists of the use of a thermoplastic biomaterial that is heated to a molten state and deposited layer by layer to manufacture the desired 3D structure (Tamay et al., 2019). FDM is simple, is cost effective and does not require the use of chemical solvents during the printing process (Tamay et al., 2019).

Scaffolds can be solid or porous, and pore size may be a determinant factor that can influence cell response because it is associated with the distribution of nutrients (Loh and Choong, 2013; Xing et al., 2019) and affects the scaffolds’ mechanical properties (Velioglu et al., 2019). The ideal pore size for maintaining cellular activities and induce specific cell response is controversial and highly variable in literature according to the cell type or response expected (Murphy and O'Brien, 2010; Loh and Choong, 2013; Abbasi et al., 2020). Nevertheless, it is crucial to guarantee the interconnectivity of the pores to allow adequate nutrient distribution and cell infiltration (Gregor et al., 2017).

The strategy of combining biomaterial scaffolds with cells can be directed to various tissue injuries depending on the cell types applied. One of the most explored cell types in tissue engineering is the mesenchymal stromal cell (MSC), due to its differentiation potential, various isolation sources, low immunogenicity and high immunomodulatory potential (Li et al., 2019; Cun and Hosta-Rigau, 2020; Wang et al., 2021). Along with MSCs, endothelial cells and their progenitors, such as the expanded CD133^+^ cell, are also crucial to tissue engineering due to their angiogenic potential (Bongiovanni et al., 2014) and paracrine activity (Angulski et al., 2017). The interaction between MSCs and scaffolds made of PLA, and how cell behavior can change according to the scaffolds’ architecture and composition have been previously explored. (Gupte et al., 2018; Persson et al., 2018; Teixeira et al., 2019). On the other hand, the literature regarding the use of 3D PLA scaffolds and endothelial progenitors, as expanded CD133 + cells, is scarcer (Islami et al., 2018).

Even though the putative use of stem/progenitor cells in regenerative medicine has been mainly associated with their differentiation capacity, the paracrine effect of these undifferentiated cells have recently gained much attention. Thus, we 3D printed PLA scaffolds with two different pore sizes to culture human adipose-derived mesenchymal stromal cells (hASCs) and expanded human CD133^+^ cells and analyze how they would behave in terms of adhesion, proliferation and immunophenotypic profile, and hence, evaluate the potential of this 3D culture for applications in tissue engineering. As we initially aimed to study the behavior of hASCs and CD133 + cells in the PLA scaffolds without favoring a specific differentiation path, we used 3D scaffolds with larger pore sizes (>500 um) to favor initial cell adhesion and nutrient distribution.

## Materials and Methods

### 3D Printed Scaffolds

PLA filament (1.75 mm) was purchased from Shenzhen Esun Industrial Co., Ltd. (Shenzhen, China). Flat-shaped PLA discs and square-shaped parallelepiped PLA scaffolds were printed in a desktop Anet A8 FDM 3D printer with an extruder diameter of 0.4 mm (precision parameters: Z = 0.004 mm; XY = 0.012 mm, and printing accuracy = 0.1–0.2 mm). The scaffolds measured 10 × 10 x 3 mm (LxWxH) and had infill densities of 25% (S25) and 40% (S40). The disc framework had an infill density of 100% and a diameter of 3 cm. For all prints, the speed was set at 30 mm/s. The extrusion and bed temperature were set at 200 and 55°C, respectively.

### Scaffold Characterization

Micro-architectures and pore size of the PLA scaffolds were examined using scanning electron microscopy (SEM). The samples were gold coated and analyzed on a JEOL JSM6010 PLUS-LA (JEOL Ltd., Tokyo, Japan) scanning electron microscope. Mean pore size was determined by measuring 20 pores from each scaffold (S25 and S40). For mechanical compressive strength characterization, a total of six scaffolds (*n* = 3/group) were tested using an EMIC DL10000 universal testing machine EMIC DL10000 (Instron, High Wycombe, United Kingdom) equipped with TESC software under a compression rate of 1.3 mm/min using a 500 N load cell. Elastic moduli were determined from the slope of the initial linear portion of the stress-strain curves obtained. The compressive strength data for each set of specimens were achieved by an average of three measurements and expressed as mean ± standard deviation (SD).

### Isolation and Culture of hASCs and CD133^+^ Cells

This study was performed following the guidelines for research involving human subjects in accordance with the Declaration of Helsinki and following the protocols and procedures for safe working in the BSL-2 laboratories of Instituto Carlos Chagas (Fiocruz Paraná). Adipose tissue (derived from liposuction surgery) and umbilical cord blood samples were collected once donors had provided their signed informed consent. This study was reviewed and approved by the Research Ethics Committee of Pontifícia Universidade Católica do Paraná and Fundação Oswaldo Cruz, Brazil (approval numbers 1366 and 419/07, respectively).

hASCs were isolated from adipose tissue obtained from one otherwise healthy female donor (BMI = 27.03) who had undergone liposuction surgery. The isolation protocol followed previously published procedures (Rebelatto et al., 2008; Marcon et al., 2020). In short, the tissue was first washed with sterile phosphate-buffered saline (PBS) and then digested with collagenase type I (Gibco^®^, Invitrogen^®^, Carlsbad, CA, United States) for 30 min at 37°C, 5% CO_2_ and under constant agitation. The lipid-enriched phase was then separated and the bottom phase was filtered through a 100 µm mesh filter (BD Bioscience) and centrifuged at 950 x g for 10 min. The supernatant was discarded and the cells were resuspended and treated for 10 min with a hemolytic buffer (0.83% ammonium chloride, 0.1% sodium bicarbonate and 0.04% EDTA). The cells were centrifuged at 150 x g for 10 min, resuspended in PBS, filtered through a 40 µm mesh filter (BD Bioscience) and plated in culture flasks at a density of 2x10^3^ cells/cm^2^ in DMEM F-12 (Dulbecco’s Modified Eagle Medium–Gibco^®^) supplemented with 10% fetal bovine serum (FBS), 1% (v/v) l-glutamine (200 mM) (Thermo Fisher, Waltham, MA, United States), 100 U/ml penicillin and 100 µg/ml streptomycin (Sigma-Aldrich, Saint Louis, MO, United States). The medium was changed twice a week and when the cells reached 70–80% confluence they were replated in DMEM supplemented with 10% FBS, 1% (v/v) l-glutamine (200 mM) (Thermo Fisher, Waltham, MA, United States), 100 U/ml penicillin and 100 µg/ml streptomycin (Sigma-Aldrich, Saint Louis, MO, United States). The cells’ identity was confirmed through flow cytometry immunophenotyping and evaluation of adipogenic and osteogenic differentiation potential (Dominici et al., 2006) prior to the experiments. All experiments were performed with cells passaged five to seven times.

Umbilical cord blood-derived CD133^+^ cells were purified and expanded as previously described (Senegaglia et al., 2010; Angulski et al., 2017). Mononuclear cells (MNCs) were isolated from human umbilical cord blood (HUCB) collected from fresh placentas with the umbilical cord still attached. MNCs were isolated first diluting the cells 1:2 (v/v) in Iscove’s Modified Dulbecco’s Medium (IMDM) (Invitrogen Life Technologies^®^, Carlsbad, CA, United States). The cells were then centrifuged for 30 min at 400 x g using Ficoll-Hypaque density gradients (Sigma-Aldrich, Saint Louis, MO, United States) and then were washed three times in IMDM. CD133^+^ cells were selected using CD133 Microbead human lyophilized kit (Miltenyi Biotech, Bergisch-Gladback, Germany), according to the manufacturer’s protocol. The purified cells were plated at a density of 1x10^5^ cells/cm^2^ in culture flasks containing Endothelial Cell Basal Medium (EBM-2) (Lonza, Basel, Switzerland) supplemented with EGM®-2 MV Microvascular Endothelial SingleQuots^®^ kit (Lonza, Basel, Switzerland). The culture medium was changed every three to 4 days until the cells were 70–80% confluent. Cell monolayers were dissociated using a solution of 0.25% of trypsin-EDTA and replated at a density of 1.3x10^4^ cells/cm^2^. All the experiments were performed with cells passaged six to eight times, when they are termed expanded CD133^+^ cells.

### Scaffold Cytotoxicity Evaluation

The potential cytotoxicity of the PLA scaffolds in culture was evaluated by direct contact assay according to the International Organization of Standardization 10,993–5 guidelines (ISO, 2009). hASCs and expanded CD133^+^ cells were plated at a density of 2x10^3^ cells/cm^2^ and 3x10^4^ cells/cm^2^, respectively, on a 24-well plate and cultured for 24 h with the supplemented medium specific for each cell type (as described in 2.3). Then, the medium was discarded and in the positive control group it was added a solution of 100 µg/ml of sodium dodecyl sulfate (SDS) in supplemented medium. For the negative control group, supplemented medium was used. For the test groups a piece of PLA scaffold was deposited on the top of the cells (three independent scaffolds were used for each cell type), which were cultured in supplemented medium. After 48 h of incubation (37°C, 5% CO_2_), cell morphology was analyzed on a Nikon Eclipse TE300 (Nikon Corporation, Tokyo, Japan) inverted microscope. Ten images of each well were obtained using the same magnification (100x).

PLA cytotoxicity was further evaluated by a neutral red uptake (NRU) assay. PLA extracts were obtained following the ISO 10993/12 (ISO, 2012) guidelines. Briefly, after the disinfection process (described below), pieces of the scaffolds weighing 0.1 g each were immersed in 1 ml DMEM, 2% l-glutamine (200 mM) (ThermoFisher, Waltham, MA, United States), 100 U/ml penicillin and 100 µg/ml streptomycin (Sigma-Aldrich, Saint Louis, MO, United States) and incubated at 37°C under slow agitation for 24, 48, 72 and 96 h. After the incubation, the PLA fragments were discarded and only the medium containing the PLA extract was used. The NRU assay was performed in duplicate and followed the Organization for Economic Co-operation and Development (OECD) guideline 129 (OECD, 2010). For the tests, BalB/c3T3 cells were cultured for 48 h with the PLA extracts or with fresh DMEM, 2% (v/v) l-glutamine (200 mM) (Thermo Fisher, Waltham, MA, United States), 100 U/ml penicillin and 100 µg/ml streptomycin (Sigma-Aldrich, Saint Louis, MO, United States) (negative control). After 48 h of culture, the cell viability was evaluated by the NRU assay following the OECD guidelines and as previously described (Abud et al., 2015). The relative growth rate (RGR) was calculated as follows: RGR = cell viability in the test group/cell viability in the control group.

A lactate dehydrogenase enzyme (LDH) assay was performed using CytoTox 96^®^ Non-Radioactive Cytotoxicity Assay kit (Promega, Madison, WI, United States). The cells were cultured on the scaffolds (3D) and on culture plates (2D) for 48 h and 7 days. The culture media were harvested and prepared for analysis according to the manufacturer’s protocol. The LDH concentration was determined by measuring the optic density of the resulting solutions at 490 nm using a Synergy H1 Hybrid Multiplate Microplate Reader (Biotek^®^, Winooski, VT, United States).

### Cell Seeding on the Scaffolds

Prior to cell seeding, the scaffolds were disinfected with ethanol 70% for 40 min, washed twice with sterile PBS and kept in PBS and 1% (v/v) PS (100 U/ml) at 4°C until cell seeding. The cells were first cultured up to 70–80% confluence on culture flasks and then were dissociated using 0.05% trypsin-EDTA and counted on a hemocytometer. A solution containing the ideal number of cells was prepared using the specific basal cell culture medium for each cell with 5% FBS and 1% (v/v) PS (100 U/ml). Then, the cells were seeded on the scaffolds using a syringe technique described by Fairag et al. (2019). Briefly, each scaffold was placed inside a syringe connected to a four-way stopcock and the medium was added until the scaffold was completely submerged. The plunger was inserted and the syringe was flipped slowly. Once the scaffold was at the bottom of the syringe, the plunger was carefully moved upwards, allowing the cells to come into contact with every surface of the scaffold-including the inside of the pores. The syringe was then placed inside the incubator (37°C and 5% CO_2_) and flipped 90° clockwise every 30 min for 2 h. After allowing the cells to attach to the scaffolds, the medium was discarded and the scaffolds were placed in a 24-well plate containing 600 µl of fresh supplemented medium and kept in the incubator. The medium was changed every 2 days.

### Cell Adhesion Monitoring

Cell adhesion was evaluated through fluorescence microscopy (FM) and SEM. The scaffolds were washed once in PBS to remove the culture medium and cells that had not completely adhered. For FM assays, the cells on the scaffolds were fixed with a solution of 4% paraformaldehyde (PFA) for 20 min. After fixation, the scaffolds were cut vertically in four parts and submerged in DAPI staining solution (1 µg/ml) for 15 min in the dark at room temperature. Then they were washed twice with PBS for 10 min and analyzed on a LEICA AF6000 (LEICA Microsystems, Wetzlar, Germany) inverted fluorescence microscope.

SEM assays were used to further evaluate cell adhesion and cell morphology on the scaffolds. After washing with PBS, the cells on the scaffolds were fixed with Karnovsky fixing solution (2.5% glutaraldehyde; 4% PFA; 0.1 M sodium cacodylate buffer solution) for 1 h at room temperature. The scaffolds were then cut vertically into four parts and the post-fixation process was carried out for 45 min with a solution of 1% of osmium tetroxide (in 0.1 M sodium cacodylate buffer). The samples were washed and then dehydrated with growing concentrations of ethanol, submitted to critical point drying, coated with gold and analyzed using a JEOL JSM6010 PLUS-LA (JEOL Ltd., Tokyo, Japan) scanning electron microscope.

### Detachment of the Cells From the Scaffolds

The scaffolds were first washed with a balanced saline solution free of calcium and magnesium (BSS-CMF) for 5 min under slow agitation and then incubated at 37°C and 5% CO_2_ with a 0.25% trypsin-EDTA solution for 4 min. Trypsin was inactivated with an equal volume of culture medium and the scaffolds were collected and centrifuged (700 x g, 30 s) to remove the trypsin and the cells that were trapped inside the pores. Both solutions were then centrifuged (700 x g, 5 min) and the cells were resuspended and counted on a hemocytometer.

### Cell Cycle Assay

Cell cycle was analyzed after 48 h and after 7 days of culture. Cells were dissociated from the scaffolds using the method previously described and resuspended in an ice-cold solution of 70% ethanol in PBS and then incubated for 1 h and 30 min at 4°C. After fixation, the cells were washed with PBS and centrifuged for 5 min at 700 x g. The supernatant was discarded and the cells were resuspended in PBS. An equal volume of 2X staining solution (3.4 mM Tris HCl pH 7.4; 0.1% Nonidet P40; 700 U/l RNase A DNase-free; 10 mM NaCl; 30 µg/ml propidium iodide) was added and the cells were incubated for 10 min in the dark at room temperature. After centrifugation, the supernatant was discarded and the cells were resuspended in 200 µl of PBS for flow cytometry analysis. Approximately 10,000 events for each sample were acquired with a FACSCanto II flow cytometer (BD Biosciences) and the analysis was performed using FlowJo software version 10.7.1. In parallel, cells were also cultured in culture flasks, to be used as control (control 2D), and on the PLA discs. The preparation of these samples, data acquisition and analysis followed the same protocol.

### Proliferation Assay

The cells were cultured for 48 h on the S40 and then were incubated with a solution of 10 µM EdU (in culture medium) for 5 h. Then, the cells were dissociated from the scaffolds, washed, fixed and stained with the Click-iT^®^ EdU Alexa Fluor^®^ 647 Flow Cytometry Assay Kit (Molecular Probes, Thermo Fisher Scientific) following the manufacturer’s instructions. Approximately 10,000 events were acquired with a FACSCanto II flow cytometer (BD Biosciences) and the analysis was performed using FlowJo software version 10.7.1. In parallel, cells were also cultured in culture flasks (2D) for 48 h for the controls with and without the EdU incubation step. The preparation of these samples, data acquisition and analysis followed the same protocol.

### Immunophenotypic Profiling

After 7 days of culture, cells were dissociated from the scaffolds and resuspended in 3 ml of PBS/bovine serum albumin (BSA) 1% and left in ice for 1 h. They were subsequently centrifuged at 700 x g for 5 min and resuspended in a PBS/BSA 1% solution containing the antibodies adequately diluted. The hASCs were labeled with the following antibodies: FITC-conjugated anti-CD90 (BioLegend, San Diego, CA, United States) and anti-CD34 (E-Bioscience, Carlsbad, CA, United States); APC-conjugated anti-CD73 and anti-HLA-DR (E-Bioscience, Carlsbad, CA, United States); PE-conjugated anti-CD105 (E-Bioscience, Carlsbad, CA, United States) and anti-CD140b (BD, San Diego, CA, United States). For the expanded CD133^+^ cells, the antibodies used were: FITC-conjugated anti-CD34 and anti-CD31 (E-Bioscience, Carlsbad, CA, United States); APC-conjugated anti-CD45 (E-Bioscience, Carlsbad, CA, United States); PE-conjugated anti-CD133, anti-CD105 (E-Bioscience, Carlsbad, CA, United States) and anti-CD146 (BD Biosciences, San Diego, CA, United States); Alexa Fluor^®^ 647-conjugated anti-CD309 (BD Biosciences, San Diego, CA, United States). For the vWF labeling, the rabbit anti-human vWF (ThermoFisher, Waltham, MA, United States) was used as the primary antibody and followed by Alexa Fluor^®^ 488 anti-rabbit (Invitrogen, Carlsbad, CA, United States). Mouse IgG antibodies (FITC, APC and PE) (BD, San Diego, CA, United States) were used as negative controls. The cells were incubated with the antibodies for 1 h at 4°C and then washed with PBS 1X. After centrifugation, the cells were fixed with PFA 4% for 10 min and in sequence were washed with PBS 1X, centrifuged and resuspended in PBS 1X. For the vWF labeling, the cells were first fixed with a solution of PFA 4%, permeabilized with a solution of PBS/Triton X-100 0.5% for 30 min and then incubated with the solution of PBS/BSA 1% containing the antibody adequately diluted. Approximately 10,000 events for each cell type were acquired with a FACSCanto II flow cytometer (BD Biosciences) and the data were analyzed using FlowJo software version 10.7.1.

### *In vitro* Angiogenesis Matrigel Assay

Expanded CD133^+^ cells were cultured on the S40 for 7 days and then were dissociated and plated on a Matrigel^®^ (Corning Inc., Corning, NY, United States) coated 96-well plate at a density of 3x10^4^ cells/well. For the control group, cells obtained from culture flasks were used. Both groups (3D and 2D) were cultured with fresh media. To determine the angiogenic potential of the secretome from 3D cultures, the conditioned medium (CM) was collected from the last 48 h of 3D cultures and used on the assay (cells were derived from culture flasks and this group was identified as 2D + 3DCM). Capillary formation was analyzed in six, 12 and 24 h using a ZEISS Primovert inverted microscope, but only the results for 12 h were considered because after 6 h there was no tube formation and after 24 h their structures were already compromised. One image capturing entirely each of the wells was recorded and the number of nodes and tubule-like structures formed was manually counted. Nodes were considered as the intersections of three ramifications (the ramifications which were on the corners of the images and, therefore, weren’t entirely visible, were not counted). Each assay was conducted with three experimental replicates. Variations of this protocol were previously tested comprising 1x10^4^, 2x10^4^ and 3x10^4^ cells/well. There was no tube formation with the lowest concentration of cells, and the other two concentrations allowed the tube formation. However, with 2x10^4^ cells/well the number of tubes was highly variable among the replicates. For this reason, we selected the concentration of 3x10^4^ cells/well to perform this assay.

### Statistical Analysis

Differences between groups were determined using one-way analysis of variance with ANOVA tests when there were at least three groups to compare. Unpaired student’s t-test was conducted when there were only two groups to compare. Significance between groups was established for *p* ≤ 0.05. The analyses were performed using GraphPad Prism software version 8.3.0.

## Results

### Scaffold Characterization Shows Rough Surfaces and Different Elastic Moduli

**FIGURE 1:**
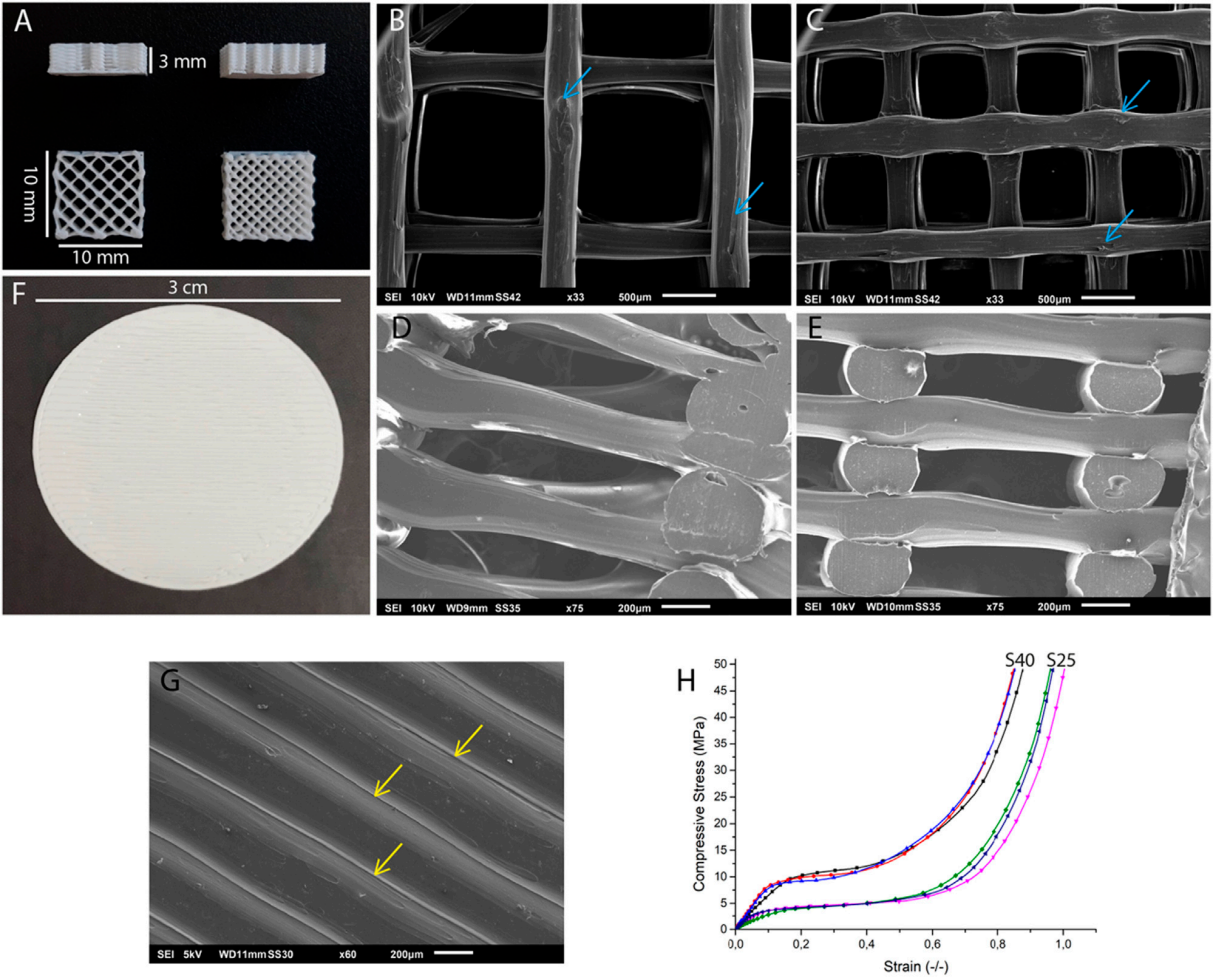
Structural and mechanical characterization of the 3D printed PLA scaffolds. **(A)** Side view of the 3D scaffold and top views of the S25 (left) and the S40 (right). SEM images of the **(B)** surface of the S25, **(C)** S40, **(D)** internal structure of the S25 and **(E)** S40. The blue arrows indicate the rough areas on the scaffolds. **(F)** PLA disc. **(G)** SEM image of the surface of the PLA disc. Yellow arrows indicate the PLA filaments forming the ruffled surface of the disc. **(H)** Mechanical testing of the PLA scaffolds. Young modulus was calculated as the slope of the linear portion of the compressive/strain curve.

The macroscopic structure of the parallelepiped-shaped scaffolds is shown in Figure 1A. SEM images showed that the surface of both scaffolds was uneven (Figures 1B–E). The S25 and S40 had a mean pore size of 1.27 ± 0.059 mm and 0.700 ± 0.023 mm, respectively. The PLA discs had no pores (Figure 1F) and just as the 3D scaffolds, their surface was not smooth. The disc’s surface was also ruffled due to the process of PLA deposition during 3D printing (Figure 1G).

The Young’s moduli calculated through the stress-strain curves (Figure 1H) obtained in the compression test were 38.90 ± 11.98 MPa for the S25 and 86.51 ± 14.40 MPa for the S40.

### The Poly(Lactic Acid) Scaffolds Are Slightly Cytotoxic

**FIGURE 2:**
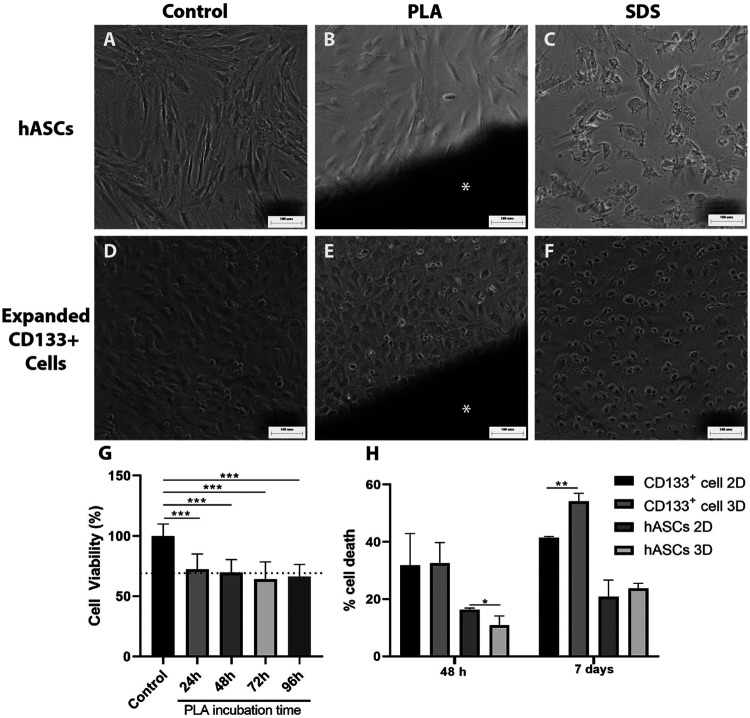
Cytotoxicity evaluation of the PLA scaffolds. Cytotoxicity was measured using direct contact **(A–F)** and NRU assays **(G)**. The images on the far left illustrate hASCs morphology **(A)** on the negative control of the direct contact assay **(B)** cultured with a fragment of the PLA scaffold and **(C)** cultured with SDS. The next three pictures illustrate the expanded CD133 + cell morphology **(D)** on the negative control of the direct contact assay **(E)** cultured with a fragment of the PLA scaffold and **(F)** cultured with SDS. Scale bar = 100 μm *PLA fragment **(G)** Cytotoxicity of the PLA scaffold observed on the NRU assay. BalB/c3T3 cells were cultured for 48 h with PLA extracts collected after 24, 48, 72 and 96 h of incubation. Cytotoxicity is represented by cell viability calculated as explained in the materials and methods section. The dotted line represents a RGR of 70%. One-way ANOVA analysis: ***p ≤ 0.001. *n* = 2 **(H)** PLA scaffolds cytotoxic potential evaluated by spectrophotometric LDH assay after 48 h and 7 days of culture. Student’s unpaired t-test analysis: *p ≤ 0.05 **p ≤ 0.01 (*n* = 3).

To evaluate the direct effect and possible toxicity of the PLA scaffolds on hASCs and expanded CD133^+^ cells in culture, first we performed a direct contact assay. After 48 h of cell culture in contact with the PLA fragments, we observed that hASCs and expanded CD133^+^ cells showed morphologies that were similar to their respective controls (Figures 2A,B,D,E). In contrast, these morphologies were different from those found in the cultures with SDS (Figures 2C,F), where there was cell lysis and, therefore, loss of characteristic morphology. These results indicate that the PLA scaffolds used in this work do not appear to be cytotoxic in this case.

To further investigate the possible toxicity of the PLA scaffolds we performed a NRU assay. A significant reduction in cell viability of approximately 30% relative to the control group was detected when the cells were cultured with the PLA extracts (Figure 2G). The RGR calculated was around 70% for the extracts and no statistical differences were observed between them. According to the ISO 10993/12 guidelines, these results indicate that the PLA extracts obtained from 24 to 96 h showed low cytotoxicity for the BalB/c3T3 cells. LDH assay (Figure 2H) confirmed this low cytotoxicity for the expanded CD133 + cells in a 7 days culture. However, no cytotoxic effect was observed for the hASCs. Moreover, there was a decrease of hASC death on the PLA scaffolds after a 48 h culture in comparison to the 2D culture.

### The Poly(Lactic Acid) Scaffolds Allow Cell Adhesion

**FIGURE 3:**
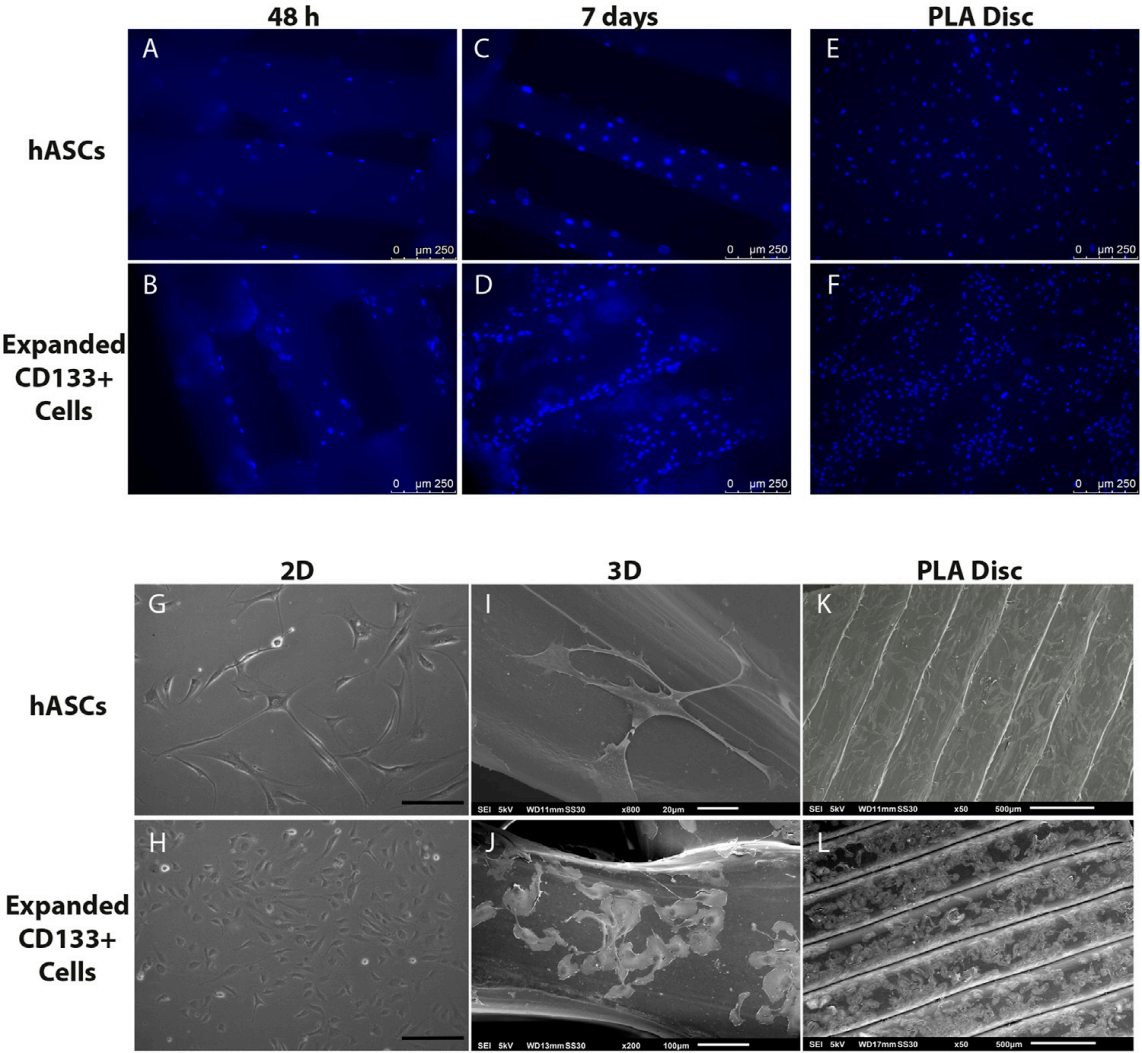
Cell adhesion and morphology on the PLA scaffolds. Top panel: nuclear staining of the cells on the scaffolds. **(A)** hASCs adhered on the 3D scaffold after 48 h of culture. **(B)** Expanded CD133^+^ cells adhered on the 3D scaffold after 48 h of culture. **(C)** hASCs adhered on the 3D scaffold after 7 days of culture. **(D)** Expanded CD133^+^ cells adhered on the 3D scaffold after 7 days of culture. **(E)** hASCs and **(F)** expanded CD133^+^ cells adhered on the PLA disc. Bottom panel: phase contrast and SEM analyses of cell morphology. **(G)** Characteristic morphology of hASCs on a 2D culture **(H)** Characteristic morphology of expanded CD133^+^ cells on a 2D culture (scale bar = 200 µm) **(I)** hASCs’ morphology on the 3D scaffold. **(J)** Expanded CD133^+^ cells’ morphology on the 3D scaffold. **(K)** hASCs’ morphology on the PLA disc. **(L)** Expanded CD133^+^ cells’ morphology on the PLA disc. The images representing the 3D scaffolds were from the cultures on the S40, which presented the same pattern as the cultures on the S25.

FM images showed that both cell types adhered homogeneously to the surface of the scaffolds and on the PLA disc (Figures 3A,B,E,F). After 7 days of culture, the number of DAPI-stained nuclei on the 3D scaffolds increased (Figures 3C,D), which was an indication of cell proliferation. SEM images further confirmed cell adhesion and also showed that, compared to a 2D culture on culture flasks (Figures 3G,H), hASCs and expanded CD133^+^ cells were able to maintain their fibroblastic and cobblestone morphology, respectively, when cultured on the 3D scaffolds (Figures 3I,J) and on the PLA disc (Figures 3K,L).

### 3D Poly(Lactic Acid)Scaffolds Differentially Influence the Cell Cycle and Proliferation

**FIGURE 4:**
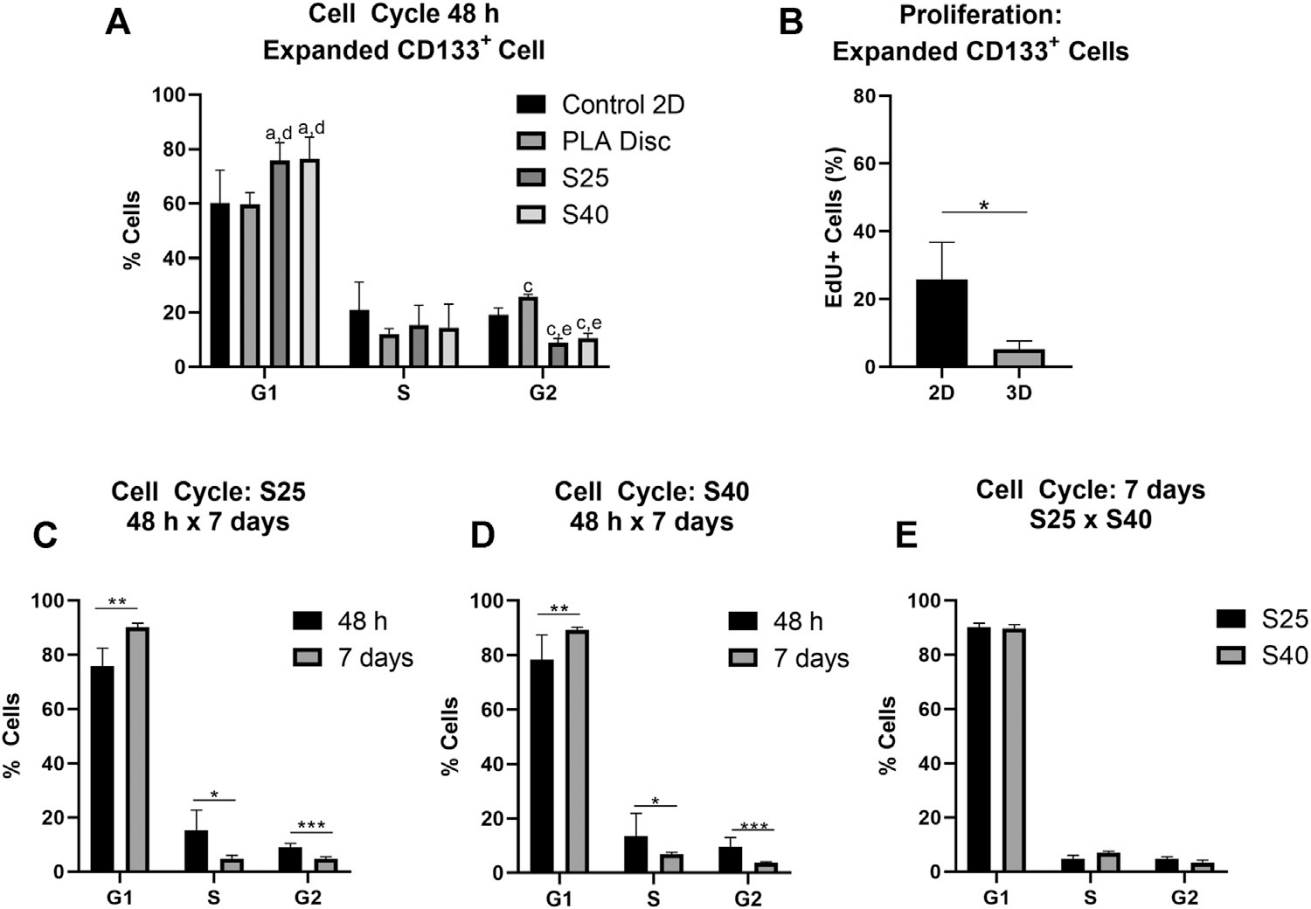
Expanded CD133+ cell cycle and proliferation after the culture on the PLA scaffolds. **(A)** Percentage of expanded CD133^+^ cells in each of the phases of the cell cycle after 48 h of culture on the scaffolds in comparison to the culture on the PLA disc and the control (Control 2D *n* = 6; PLA disc *n* = 3; S25 *n* = 8; S40 *n* = 8). Analysis by one-way ANOVA: ^a^ *p* ≤ 0.05 in comparison to the control 2D; ^c^ *p* ≤ 0.001 in comparison to the control 2D; ^d^ *p* ≤ 0.05 in comparison to the PLA disc; ^e^ *p* ≤ 0.01 in comparison to the PLA disc **(B)** Cell proliferation analysis by EdU incorporation after 48 h of culture on the 3D scaffold in comparison to the control 2D (Control 2D *n* = 2; 3D *n* = 3) **(C)** Percentage of cells in each of the phases of the cells cycle after 48 h and 7 days of culture on the S25 (S25 7 days *n* = 5) **(D)** and on the S40 (S40 7 days *n* = 4) **(E)** Comparison of the percentage of cells in each of the phases of the cell cycle after 7 days of culture on the S25 and S40. For the 3D scaffolds n represents the number of scaffolds that were analyzed. Graphs are represented by the mean ± SD. Student’s unpaired t-test analysis: *p ≤ 0.05; **p ≤ 0.01; ***p ≤ 0.001.

The effect of the 3D PLA scaffolds on the progress of the cell cycle was evaluated by comparing the percentage of cells in the different cell cycle phases of the hASCs and CD133^+^ cells cultured on 3D PLA scaffolds, PLA discs and in culture flasks (control 2D). After 48 h of culture, most expanded CD133^+^ cells were in the G1 phase of the cell cycle in all culture conditions (Figure 4A). There was no statistical difference between the percentage of cells in G1 on the control 2D and on the PLA discs, but there was an increase in the percentage of cells in the G1 phase on both 3D scaffolds (control 2D: 60.2% ± 12.14; PLA Disc: 59.7% ± 4.37; S25: 75.8% ± 6.51; S40: 76.5% ± 7.91). Compared to the 2D control, the percentage of cells in the G2 phase of the cell cycle significantly decreased on the 3D scaffolds by approximately 55%, and there was a significant increase (around 35%) on the PLA discs (control 2D: 19.2% ± 2.53; PLA Disc: 25.9% ± 0.86; S25: 8.6% ± 1.28; S 40: 10.6% ± 1.77). There was no statistical difference in cell cycle among the 3D scaffolds, meaning that the pore size did not seem to affect cell cycle in 48 h of culture. Proliferation analysis further confirmed a significant reduction of proliferating expanded CD133^+^ cells when they were cultured on the 3D scaffolds (Figure 4B).

To evaluate if pore size could influence cell behavior after a longer period of culture, we also analyzed the cell cycle of the expanded CD133^+^ cells after 7 days in culture. In this case, the percentage of cells in G1 increased relative to their levels at 48 h (S25: 90.1% ± 1.48; S40: 89.7% ± 1.29), while the percentage of cells in S (S25: 4.87% ± 1.41; S40: 6.95% ± 0.71) and G2 decreased (S25: 4.8% ± 0.76; S40: 3.38% ± 0.93) (Figures 4C,D). However, there was still no significant difference in the cell cycle between the S25 and S40 (Figure 4E).

**FIGURE 5:**
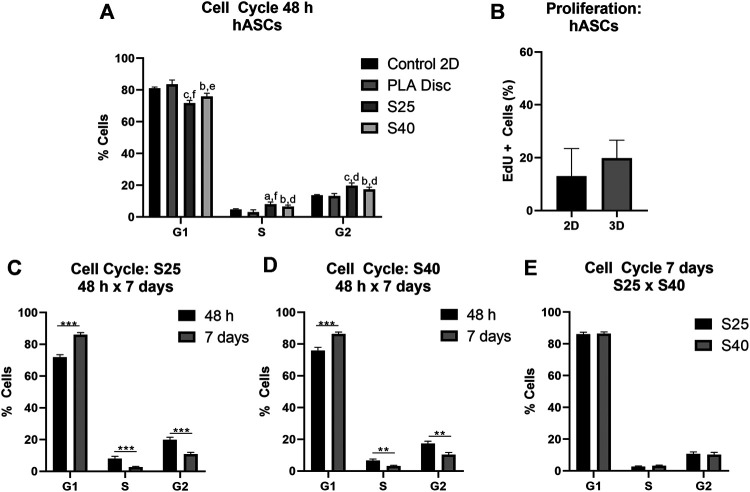
hASCs cell cycle and proliferation after the culture on the PLA scaffolds **(A)** Percentage of hASCs in each of the phases of the cell cycle after 48 h of culture on the scaffolds in comparison to the culture on the PLA disc and the control (Control 2D *n* = 4; PLA disc *n* = 3; S25 *n* = 4; S40 *n* = 4). One-way ANOVA: ^a^ *p* ≤ 0.05 in comparison to the control 2D; ^b^ *p* ≤ 0.01 in comparison to the control 2D; ^c^ *p* ≤ 0.001 in comparison to the control 2D; ^d^ *p* ≤ 0.05 in comparison to the PLA disc; ^e^ *p* ≤ 0.01 in comparison to the PLA disc; ^f^ *p* ≤ 0.001 in comparison to the PLA disc **(B)** Cell proliferation analysis by EdU incorporation after 48 h of culture on the 3D scaffold in comparison to the control (2D) (Control 2D *n* = 4; 3D *n* = 4) **(C)** Percentage of cells in each of the phases of the cells cycle after 48 h and 7 days of culture on the S25 (S25 7 days *n* = 4) **(D)** and on the S40 (S40 7 days *n* = 3) **(E)** Comparison of the percentage of cells in each of the phases of the cell cycle after 7 days of culture on the S25 and S40. For the 3D scaffolds n represents the number of groups comprising three scaffolds that was analyzed. Graphs are represented by the mean ± SD. Student’s unpaired t-test analysis: *p ≤ 0.05; **p ≤ 0.01; ***p ≤ 0.001.

As observed for the expanded CD133^+^ cells, hASCs were predominantly concentrated on the G1 phase in all conditions after 48 h of culture (Figure 5A) (Control 2D: 82.48% ± 2.36; PLA Disc: 83.50% ± 2.75; S25: 71.83% ± 1.62; S40: 75.90% ± 2.06). Nevertheless, differently than what was observed for the expanded CD133^+^ cells, there was no statistical difference between the hASCs cultured on the culture flasks and on the PLA discs in any of the phases of the cell cycle. However, there was an increase of nearly 58% in the percentage of cells in the G2 phase when they were cultured on the 3D scaffolds (Control 2D: 12.53% ± 1.84; PLA Disc: 13.23% ± 1.59; S25: 19.78% ± 1.64; S40: 17.35% ± 1.45). Proliferation analysis showed no significant difference between the 2D and 3D cultures (Figure 5B). Again, there was no significant difference in the cell cycle among the 3D scaffolds after 48 h (Figure 5A).

Similarly to the expanded CD133^+^ cells, after 7 days in culture, there was an increase in the percentage of cells in G1, a reduction in S (S25: 2,67% ± 0,42; S40: 3,18% ± 0,44) and G2 (S25: 11.0% ± 1.31; S40: 10.3% ± 1.48) (Figures 5C,D) with no significant difference between the scaffolds (Figure 5E).

### Cell Immunophenotypic Profile can Change After 3D Culture

**FIGURE 6:**
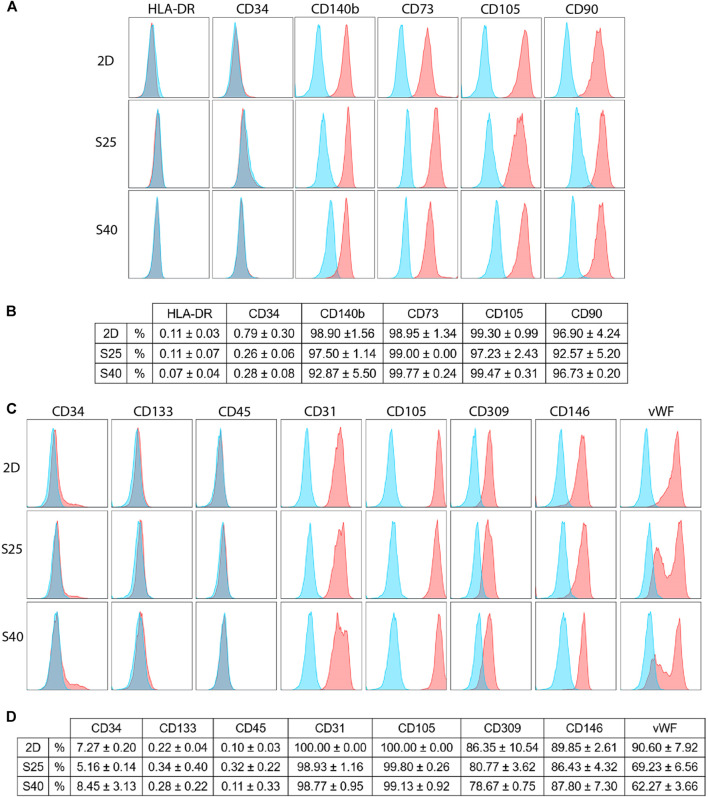
Immunophenotypic profile of the expanded CD133+ cells and the hASCs after 7 days of culture on the scaffolds in comparison to a 2D culture **(A)** Representative histograms of the hASCs immunophenotypic profile when cultured on a 2D culture flask and on the 3D scaffolds **(B)** hASCs immunophenotypic profile represented in percentage values (mean ± SD) (2D *n* = 2; S25 *n* = 3; S40 *n* = 3). For the 3D scaffolds n represents the number of groups comprising three scaffolds that was analyzed. One-way ANOVA analysis **(C)** Representative histograms of the expanded CD133^+^ cells’ immunophenotypic profile when cultured in a 2D culture flask and on the 3D scaffolds **(D)** Expanded CD133^+^ cells’ immunophenotypic profile represented in percentage values (mean ± SD) (2D *n* = 2; S25 *n* = 3; S40 *n* = 3). For the 3D scaffolds n represents the number of scaffolds analyzed. One-way ANOVA analysis. % = percent of parental population considering live/singlet gated cells.

To investigate if the scaffolds could trigger changes on the cells’ immunophenotype, flow cytometry analyses were carried out using a set of markers for each cell type. After 7 days of culture, the hASCs showed high expressions of CD90, CD105, CD73 and CD140b, while keeping the expressions of CD34 and HLA-DR low. There were no differences in their immunophenotype in comparison to the control (Figures 6A,B), suggesting that these cells were able to maintain their undifferentiated profile after being cultured on the scaffolds.

The expanded CD133^+^ cells cultured on the scaffolds presented, for the most part, a similar immunophenotype profile to their respective control, showing low expression of CD34 and CD45 and high expression of endothelial markers, such as CD31. In all the groups, the expression of CD133 was low. However, in comparison to the control, there was a significant reduction of vWF when the cells were cultured on the S25 (*p* = 0.02) and S40 (*p* = 0.008). Interestingly, besides this reduction we also noticed a change in its labelling pattern. While in the control we observed only one population of cells showing a high fluorescence intensity for vWF, on the 3D scaffolds there were at least two distinguished populations with high and low fluorescence intensity (Figures 6C,D).

### Culture on 3D Poly(Lactic Acid) Scaffolds Affects the Expanded CD133^+^ Cells’ Angiogenic Potential

Since vWF is associated with angiogenesis, playing an important role in controlling the formation of new blood vessels, we investigated whether the reduction in vWF observed in the immunophenotypic profiling could affect the angiogenic potential of the expanded CD133^+^ cells. Because there were no differences in the cell phenotype when they were cultured on the scaffolds with two different pore sizes, we selected only the cells cultured on S40 for this angiogenic assay.

**FIGURE 7:**
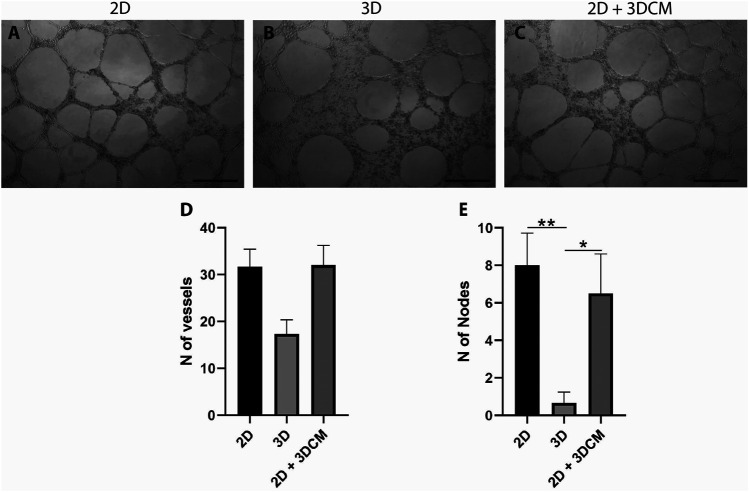
Capillary-like tubule formation assay of expanded CD133+ cells cultured for 12 h on a Matrigel layer. Representative image of tubule-like formation for each group **(A)** control **(B)** 3D cells and **(C)** 3D secretome. Scale bar = 500 µm. Analysis of the number of **(D)** vessels and **(E)** nodes formed after 12 h. Graphs are represented by the mean ± SD. One-way ANOVA: *p ≤ 0.05; **p ≤ 0.01 (2D *n* = 3; 2D + 3DCM *n* = 2; 3D *n* = 3).

After 12 h of culture on the Matrigel layer, the average number of tubule-like structures formed by the cells derived from the 3D culture was lower in comparison to the control cells (2D) (Figures 7A,B), albeit not statistically significant (Figure 7D). Furthermore, vWF is an intracellular factor which is secreted during angiogenesis (Starke et al., 2011). Thus, we also investigated if the secretome of the 3D culture could modulate the formation of tubule-like structures. In this case, compared to the control, the cells cultured with the conditioned media (2D + 3DCM) behaved in a similar manner, forming the same number of structures (Figures 7C,D). We also analyzed the formation of tubule-like structures after 6 and 24 h of culture, but there were no significant differences between either of the groups (data not shown).

In terms of number of nodes, the cells derived from the 3D culture formed significantly fewer nodes than the 2D and 2D + 3DCM groups (Figure 7E).

## Discussion

The combination of stem cells and biomaterial scaffolds is a classical approach to tissue engineering and has been extensively studied (Oshima et al., 2009; Liang et al., 2019). However, although there are many studies exploring the potential of MSCs (Lobo et al., 2015; Jakus et al., 2016; Diomede et al., 2018; Theodoridis et al., 2020) few have assessed the use of endothelial progenitor cells (Islami et al., 2018) in association with scaffolds, notably with the expanded CD133^+^ cells, which represent an uncomplicated source of cells with therapeutic value.

Every biomaterial has advantages and disadvantages, and its structures and surfaces can be modified to meet the specific needs of each tissue (Singhvi et al., 2019; Carvalho et al., 2020). PLA is a well-known biomaterial that is also widely used for these applications because it is versatile, cheap and easily manipulated (Singhvi et al., 2019; Casalini et al., 2019). Furthermore, its biocompatibility is widely known and has been well reported in literature (Silva et al., 2018; Carvalho et al., 2020). Nevertheless, variations in the processing of PLA scaffolds, such as the sterilization method used, can affect its use in tissue engineering (Savaris et al., 2017).

Thus, we began by evaluating the PLA scaffolds’ cytotoxicity using a direct contact assay and NRU assay. The direct contact assay did not accuse toxicity for either of the cell types used. Meanwhile the NRU assay, which evaluated the toxicity of possible degradation products, showed low cytotoxicity for BalB/c 3T3 cells. Although these cells are used as a model in some assays, they are not always a good model for mammalian cells and can be considered less resistant than hASCs (Abud et al., 2015). In this regard, Grémare and colleagues demonstrated, using an approach similar to ours, that media extracts of 3D printed PLA scaffolds did not significantly affect either the metabolic activity or the cell viability of human bone marrow stromal cells (Grémare et al., 2017). LDH activity assay, a commonly used test to assess the cytotoxicity of 3D scaffolds (Dinescu et al., 2013; Kumar et al., 2020), demonstrated that the scaffolds created an appropriate environment for the hASCs survival throughout cultivation. Indeed, we observed less cell death after 48 h on the scaffolds than on a standard 2D culture. Conversely, even though they also did not show cytotoxic effects for the expanded CD133+ cells in a 48 h culture, after 7 days there was an increase of cell death in comparison to the 2D culture, which might indicate that the environment became cytotoxic as the cell population expanded.

Regarding cell adhesion, fluorescence microscopy and SEM images showed that cells were efficiently seeded and had spread throughout the mesh of the PLA scaffolds. Both cell types also maintained their proper morphology and were able to survive, indicating that the environment created by the scaffolds was suitable for cell maintenance. However, even though we cultured cells in a 3D system, SEM images showed that the cells adhered along the PLA filaments in a bidimensional manner due to the difference in the proportion between pore and cell sizes.

One crucial aspect of PLA scaffolds that can affect cell behavior is their stiffness (Zonderland and Moroni, 2021), which can vary according to the production technique used and the scaffold’s final structure (Song et al., 2017; Liang et al., 2019). Rosenzweig et al. (2015) 3D printed PLA scaffolds of 1 MPa stiffness while Velioglu et al. (2019) constructed scaffolds with stiffness of up to 640 MPa demonstrating the effect of pore size on PLA scaffold stiffness. In this work, the S25 and S40 scaffolds exhibited elastic moduli of 38.90 ± 11 MPa and 86.51 ± 14.40 MPa, respectively. Comparing these values with data from the literature on the stiffness of living tissues (Guimarães et al., 2020), this characteristic should allow these scaffolds to be used in applications for bone regeneration, which is expected, since PLA scaffolds are commonly used for bone and cartilage applications (Haaparanta et al., 2014; Rosenzweig et al., 2015; Gregor et al., 2017; Söhling et al., 2020).

Besides mechanical characteristics, another variable that can influence cell response is the scaffold’s topography (Wang et al., 2021). In this case we observed that the rugged surface of the PLA discs seemed to influence how the expanded CD133^+^ cells organized themselves on the disc, causing them to follow the orientation of the PLA filaments. Heath and colleagues (2010) observed a similar behavior with umbilical vein endothelial cells (HUVECs) cultured on fibrous scaffolds. The authors observed that the cells followed the direction of the fibers and showed a more elongated morphology. They suggest that this behavior is expected since cells organize themselves that way in biological tissues to form blood vessels with elongations in their morphologies due to the shear rate of blood flow (Heath et al., 2010). In our case, one of the reasons why the cells’ morphologies remained unchanged could have been the lack of culture medium flow, but the FM and SEM images showed the cells’ preference to organize themselves following the direction of the PLA filaments.

For the hASCs, though, we did not observe differences in how the cells distributed themselves along the PLA disc compared to the 3D scaffolds, and there were no other differences in cell behavior in the analyzed parameters. However, some studies have shown the influence of scaffold topography on the paracrine activity of these cells (Leuning et al., 2018; Simitzi et al., 2020). Su and colleagues (2017) showed that when hASCs were cultured on fibrous scaffolds, the cells produced more anti-inflammatory and pro-angiogenic cytokines than in a 2D culture. Also, different fiber orientations resulted in different quantities and types of cytokines produced (Su et al., 2017).

In the EdU incorporation assay, we observed that the proliferative activity of hASCs cultured on the 3D scaffolds was highly variable, which could have been driven by the unsynchronized cell populations. Still, we observed that the S25 and S40 stimulated the cell cycle progression of hASCs in the first 48 h of culture, which is consistent with previous studies that showed that PLA scaffolds can stimulate the proliferative activity of fibroblasts (Korpela et al., 2013) and bone marrow MSCs (Teixeira et al., 2019). On the other hand, when the expanded CD133^+^ cells were cultured on the S25 and S40 the cells came to a halt in the G1 phase and showed less proliferative activity. Interestingly, however, on the PLA disc there was an increase of expanded CD133^+^ cells in the G2 phase. This suggests that the biomaterial and/or the disc topography stimulated cell cycle progression and that the G1 phase arrest observed on the S25 and S40 was possibly related to the 3D microenvironment created by the porous scaffolds.

For both hASCs and expanded CD133 + cells, the progression of the cell cycle slowed down after 7 days of culture relative to the first 48 h, which may be related to higher cell confluence (observed by FM images) and not necessarily to the scaffolds or the biomaterial. Moreover, there were no differences in any of the phases of the cell cycle between the cultures on the S25 and the S40 for either of the cell types. In summary, these results suggest that in terms of cell cycle and proliferation, the microenvironment created by the 3D scaffolds was advantageous to the hASCs, but discouraging to the expanded CD133 + cells. In addition, we found that the difference in the size of the pores used in this work was insufficient to create different cell responses considering the parameters analyzed.

Lastly, we investigated if the cells could maintain their immunophenotypic profiles after being cultured on the scaffolds for 7 days. hASCs showed high expression of MSC markers (CD90, CD105, CD73, CD140b) without significant expression of CD34 and HLA-DR, indicating that they preserved their undifferentiated profile (Dominici et al., 2006; Bühring et al., 2007). These results are consistent with findings in the literature that state that MSCs are only able to differentiate on PLA if they are cultured with induction medium (Fairag et al., 2019) or if PLA is coated or combined with a biomaterial that is capable of inducing differentiation (Persson et al., 2018; Teixeira et al., 2019).

The expanded CD133^+^ cells showed low expression of CD133 in all conditions, a characteristic of mature endothelial cells. In addition, they also showed high expression of other endothelial markers such as CD309, CD105, CD146 and CD31, and low expression of hematopoietic markers CD34 and CD45, reinforcing their endothelial profile in all culture conditions (Ingram et al., 2004; Senegaglia et al., 2010; Bongiovanni et al., 2014). However, we observed a significant reduction in the labeling of vWF when the cells were cultured on the scaffolds, which could indicate endothelial stimulation. Because vWF is associated with angiogenesis (Starke et al., 2011), we used the cells from the scaffolds to perform an angiogenesis assay to evaluate if this reduction was sufficient to affect the cells’ angiogenic potential. We observed a non-significant reduction in the number of tubule-like structures formed by the 3D cells, but a significant reduction in the number of nodes formed by these cells. This may indicate that, even though the cells were still capable of forming tubule-like structures, the vessel network was less complex. The control and the 2D + 3DCM groups behaved similarly in both tubule-like and nodes formation, which indicates that the secretome of the expanded CD133^+^ cells cultured in porous PLA scaffolds does not modulate angiogenesis, at least in the conditions analyzed. Finally, because the main goal of this work was to evaluate the behavior of the cells on the scaffolds considering their ability to survive (adherence, proliferation, and ability to maintain their undifferentiated state) and considering we did not observe changes on the hASC markers we did not conduct a functional test for these cells.

## Conclusion

The 3D PLA scaffolds created a functional environment for hASC culture, stimulating the cell cycle progression while also allowing the maintenance of their undifferentiated state. Conversely, the proliferation of expanded CD133^+^ cells was reduced and their immunophenotype changed. The data acquired in our work highlight how the same scaffold composition and architecture may lead to different cell responses depending on the cell type used. This reinforces the importance of studying basic cell behavior when we consider the combination of biomaterial scaffolds and stem/progenitor cells approach for tissue engineering.

## Data Availability

The original contributions presented in the study are included in the article/Supplementary Material, further inquiries can be directed to the corresponding authors.
